# 7-T clinical MRI of the shoulder in patients with suspected lesions of the rotator cuff

**DOI:** 10.1186/s41747-019-0142-1

**Published:** 2020-02-07

**Authors:** Andrea Lazik-Palm, Oliver Kraff, Stefan H. G. Rietsch, Mark E. Ladd, Michael Kamminga, Sascha Beck, Harald H. Quick, Jens M. Theysohn

**Affiliations:** 1 Erwin L. Hahn Institute for Magnetic Resonance Imaging, University Duisburg-Essen, Essen, Germany; 2 Department of Diagnostic and Interventional Radiology and Neuroradiology, University Hospital Essen, University Duisburg-Essen, Hufelandstr. 55, 45147 Essen, Germany; 3 High Field and Hybrid MR Imaging, University Hospital Essen, University Duisburg-Essen, Essen, Germany; 4 Medical Physics in Radiology, German Cancer Research Center (DKFZ), Heidelberg, Germany; 5 Faculty of Physics and Astronomy and Faculty of Physics, University of Heidelberg, Heidelberg, Germany; 6 Procelsio Clinic GmbH, Essen, Germany; 7 Department of Trauma and Orthopedic Surgery, University Hospital Essen, University Duisburg-Essen, Essen, Germany; 8 Department of Orthopaedics and Orthopaedic Surgery, Saarland University Medical Center and Saarland University Faculty of Medicine, Homburg, Germany

**Keywords:** 7 Tesla, Arthroscopy, Magnetic resonance imaging, Rotator cuff, Shoulder

## Abstract

**Background:**

To evaluate feasibility and diagnostic performance of clinical 7-T magnetic resonance imaging (MRI) of the shoulder.

**Methods:**

Eight patients with suspected lesions of the rotator cuff underwent 7-T MRI before arthroscopy. Image quality was scored for artifacts, B_1_^+^ inhomogeneities, and assessability of anatomical structures. A structured radiological report was compared to arthroscopy. In four patients, a visual comparison with pre-existing 1.5-T examinations was performed.

**Results:**

Regarding image quality, the majority of the sequences reached values above the middle of each scoring scale. Fat-saturated proton density sequences showed least artifacts and best structure assessability. The most homogenous B_1_^+^ field was reached with gradient-echo sequences. Arthroscopy did not confirm tendinopathy/partial tear of supraspinatus in 5/8 patients, of subscapularis in 5/6, and of infraspinatus in one patient; only a partial lesion of the subscapularis tendon was missed. Pathologic findings of long bicipital tendon, acromioclavicular joint, glenohumeral cartilage, labrum, and subacromial subdeltoideal bursa were mainly confirmed; exceptions were one lesion of the long bicipital tendon, one subacromial bursitis, and one superior glenoid labrum anterior-to-posterior lesion, missed on 7-T MRI. Evaluating all structures together, sensitivity was 86%, and specificity 74%. A better contrast and higher image resolution was noted in comparison to previous 1.5-T examinations.

**Conclusions:**

7-T MRI of the shoulder with diagnostic image quality is feasible. Overrating of tendon signal alterations was the main limitation. Although the diagnostic performance did not reach the current results of 3-T MRI, our study marks the way to implement clinical 7-T MRI of the shoulder.

## Key points

- Magnetic resonance imaging of the shoulder with diagnostic image quality is feasible at 7 T.
- Fat-saturated proton density sequences showed least artifacts and best structure assessability.
- Pathologic findings appeared as more distinct at 7 T compared to 1.5 T.
- Readers should be aware of possible overrating of tendon signal alterations.

## Background

Magnetic resonance imaging (MRI) is the most comprehensive imaging method to visualise soft tissue pathologies of the shoulder [1, 2]. As a standard of care procedure, it is widely used at field strengths of 1.5 or 3 T and has proven high diagnostic accuracy in the diagnosis of rotator cuff tears [3]. Room for improvement exists in subtler abnormalities [4] like low grade cartilage lesions, where a reduced accuracy at both 1.5 and 3 T has been shown [5], or the classification of superior glenoid labrum anterior to posterior (SLAP) lesions [6]. Here, higher spatial resolution and better tissue contrast are desired. These demands can either be solved by extending the examination time to a certain extent or by further increasing the magnetic field strength [7]. Following the trend showed in studies comparing 1.5-T to 3-T MRI of the shoulder in the detection of partial- and full-thickness rotator cuff tears [8], we postulated that even more accuracy should be gained from further increased field strengths.

Ultra-high field (UHF), in particular 7-T, MRI systems have been established and evaluated during the last decade to approach this challenge. At more than 60 sites worldwide, a substantial quantity of investigations has shown diagnostic improvements in several parts of the body, *i.e.*, in the brain [9], the heart [10], or the hip [11] as well as the possibility to reduce the amount of injected contrast media [12, 13]. These achievements are mainly based on the higher spatial resolution and better tissue contrast at UHF [14]. So far, the Food and Drug Administration has cleared MRI of the head and knee joint at 7 T as clinical applications for the latest generation of 7-T MRI systems [15]. However, MRI of the shoulder at 7 T is still in its infancy. Challenges to overcome in 7-T shoulder MRI are in particular the peripheral location of the joint in the human body, making it difficult to place the region of interest in the centre of the magnet, and the absence of commercially provided radiofrequency (RF) coils for musculoskeletal imaging [16], resulting in the need for individual self-built solutions [17].

A literature search carried out in PubMed in April 2019 (search criteria: “shoulder” AND (“mri” OR “mr”) AND (“7 T” OR “7 T” OR “7 tesla” OR “UHF” OR ”ultra-high-field” OR “ultra high field”) identified only two studies dealing with 7-T shoulder MRI [18, 19]. Both studies focused on the development of an RF coil setup dedicated for 7 T as well as on the evaluation of shim modes and demonstrated high image quality in example *in vivo* measurements. Another study presented preliminary results for musculoskeletal imaging obtained with a multipurpose coil [20], revealing difficulties with B_1_ inhomogeneities and limited penetration depth in larger joints like the shoulder. Thus, up to now, studies regarding clinical applicability of 7-T shoulder MRI are missing. In this work, 7-T shoulder MRI is applied in a patient cohort for the first time and findings are correlated to the arthroscopy report.

## Methods

### Study population

Approval from the local institutional ethics committee was gained before the study and all participants signed informed consent. In cooperation with one particular surgeon, patients presenting with shoulder pain in an orthopaedic outpatient department were enrolled to participate in the study. Inclusion criteria were shoulder pain with suspected lesions of the rotator cuff, scheduled shoulder arthroscopy, and the ability to lie still for approximately 60 min in the bore of the magnet. Exclusion criteria were previous surgeries of the painful shoulder, claustrophobia, obesity (body mass index ≥ 30 kg/m^2^), implants in the region of the painful shoulder, and pregnancy. The general presence of implants was carefully evaluated and assessed in consensus with our local safety guidelines weather a 7-T scan was possible or not. During the duration of 7 months, eight patients matching all the criteria and willing to participate in the study were identified. They were six males and two females, aged 48.3 ± 10.0 years (mean ± standard deviation [SD]).

### Technical requirements

A whole-body 7-T research MRI system (Magnetom 7 T, Siemens Healthcare GmbH, Erlangen, Germany) equipped with an in-house built c-shaped 8-channel transmit/receive RF coil and an in-house built 7-channel receive RF coil [19] were used. Patients were placed in the supine position, head first and arms parallel to the examination table with the coils placed around the pathological shoulder. To minimise B_1_^+^ inhomogeneities with maximum signal amplitude, subject-individual phase-only RF shimming was used.

### Imaging protocol

The applied standard sequences were chosen in accordance with the protocol recommendations for shoulder imaging of the German society of radiology [21] and complemented by a range of additional sequences from other joint protocols already existing in our library [11, 22]. In total, ten sequences were included in the protocol, balancing a maximum of variety for evaluation in limited examination time. Details about the sequence parameters are given in Table 1.

**Table 1:** Sequence parameters of the 7-T shoulder MRI protocol.

Weighting/type	Orientation	Type of sequence	Fat suppression	In-plane resolution (mm^2^)	Slice thickness (mm)	Number of slices	Distance factor (%)	Acquisition time (min)	TR (ms)	TE (ms)	FOV (mm^2^)
Standard sequences											
PD	Axial	Turbo spin-echo	Fat-sat	0.43 × 0.43	2.5	30	30	4:05	4,350	30	180 × 180
PD*	Coronal	Turbo spin-echo	Fat-sat	0.43 × 0.43	2.5	23	30	4:05	4,350	30	180 × 180
T1	Sagittal	Gradient-echo	None	0.45 × 0.35	2.0	40	20	5:24	172	4.1	180 × 180
Additional sequences											
PD	Sagittal	Turbo spin-echo	None	0.40 × 0.40	2.5	30	20	3:38	4,500	35	200 × 200
PD	Coronal	Turbo spin-echo	None	0.40 × 0.40	2.5	30	20	3:15	4,500	35	200 × 200
STIR	Coronal	Turbo spin-echo	Inversion time 300 ms	0.70 × 0.70	3.0	20	20	2:03	4,500	31	180 × 180
MEDIC	Coronal	Multi-echo spoiled gradient-echo	None	0.35 × 0.35	1.5	39	50	5:21	1,000	15	180 × 180
DESS 3D	Coronal	Steady-state free precession	Water excitation	0.70 × 0.70	0.7	128	0	4:56	8	2.4	190 × 190
T1	Coronal	Gradient-echo	None	0.45 × 0.35	2.0	40	20	5:24	172	4.1	180 × 180
T1	Coronal	Turbo spin-echo	None	0.40 × 0.40	2.5	9	20	1:43	900	11	200 × 200

*3D* Three-dimensional, *DESS* Dual-echo steady state, *Fat-sat* Fat saturated, *FOV* Field of view, *MEDIC* Multi-echo data image combination, *PD* Proton density, *STIR* Short-tau inversion recovery, *TE* Echo time, *TR* Repetition time. Except for T1-weighted TSE/GRE, all sequences used parallel imaging technique 2

*According to guidelines, a double echo (PD- and T2-weighted) sequence should be used. However, as T2-imaging with adequate signal-to-noise ratio remains challenging at ultra-high fields, we decided to forego the second echo

### Image analysis

Image quality was assessed in consensus by two radiologists separately for each patient and each sequence using Likert scales [23–25]. For this issue, the presence of artifacts was rated on a 5-point scale: score 5 = excellent image quality without any artifacts; score 4 = very good image quality with hardly definable artifacts/artifacts out of region of interest (*i.e.*, pulmonary); score 3 = few artifacts without affection of diagnostic image quality; score 2 = moderate artifacts with affection of diagnostic image quality/overall reduced signal-to-noise ratio; score 1 = severe artifacts with non-diagnostic image quality. The amount of B_1_^+^ inhomogeneities was rated on a 3-point scale: score 3 = no relevant heterogeneities; score 2 = moderate heterogeneities; score 1 = severe heterogeneities. The assessability of structures was rated on a 4-point scale separately for the bone, muscle, tendons, cartilage, labrum, joint cavity, vessels/nerves: score 4 = excellent; score 3 = moderate; score 2 = fair; score 1 = not assessable. Mean values with standard deviations for all patients were calculated for each criteria and sequence. A statistical analysis was waived because of the explorative nature of the study and the small number of subjects.

Additionally, a structured report focusing on the rotator cuff [26] was generated in consensus by the same radiologists, assessing:

- Tendons (intact, tendinopathy, partial tear, full thickness tear);
- Possible fatty atrophy of the rotator cuff muscles (normal, grades 1–4 according to Goutallier classification [27];
- Possible supraspinatus atrophy (convex, plane, or concave);
- Subacromial subdeltoideal bursa (normal, effusion, thickening, or effusion and thickening);
- Shape of the acromion (flat, curved, hooked, convex);
- Acromioclavicular joint (normal, mild degenerative changes, severe degenerative changes, activated arthrosis);
- Effusion of the glenohumeral joint (yes or no);
- Cartilage (normal, thinned, transchondral defect);
- Labrum (intact, SLAP lesion, Bankart lesion, paralabral cysts);
- Bone (normal, oedema, other bone marrow signal alterations).

The quality of the report was afterwards judged in comparison with the arthroscopy report taken as the gold standard. Finally, the pathologies found on 7-T MRI were visually compared with pre-existing 1.5-T MRI examinations provided by the patients.

### Data presentation and statistical analysis

Data are presented as natural frequencies or mean ± SD. Sensitivity, specificity, and positive and negative predictive values of 7-T MRI *versus* arthroscopy were calculated according the binomial distribution using SPSS Statistics 25 (IBM, Armonk, USA).

## Results

The whole imaging protocol was applicable in seven of eight patients. In one patient, however, the acquisition of six differently weighted sequences (T1 coronal, proton density [PD] sagittal, PD coronal, PD fat-saturated [fat-sat] axial, three-dimensional [3D] multi-echo data image combination [MEDIC] coronal, and short-time inversion recovery [STIR] coronal) failed due to a technical hardware problem.

### Image quality

**Fig. 1:**
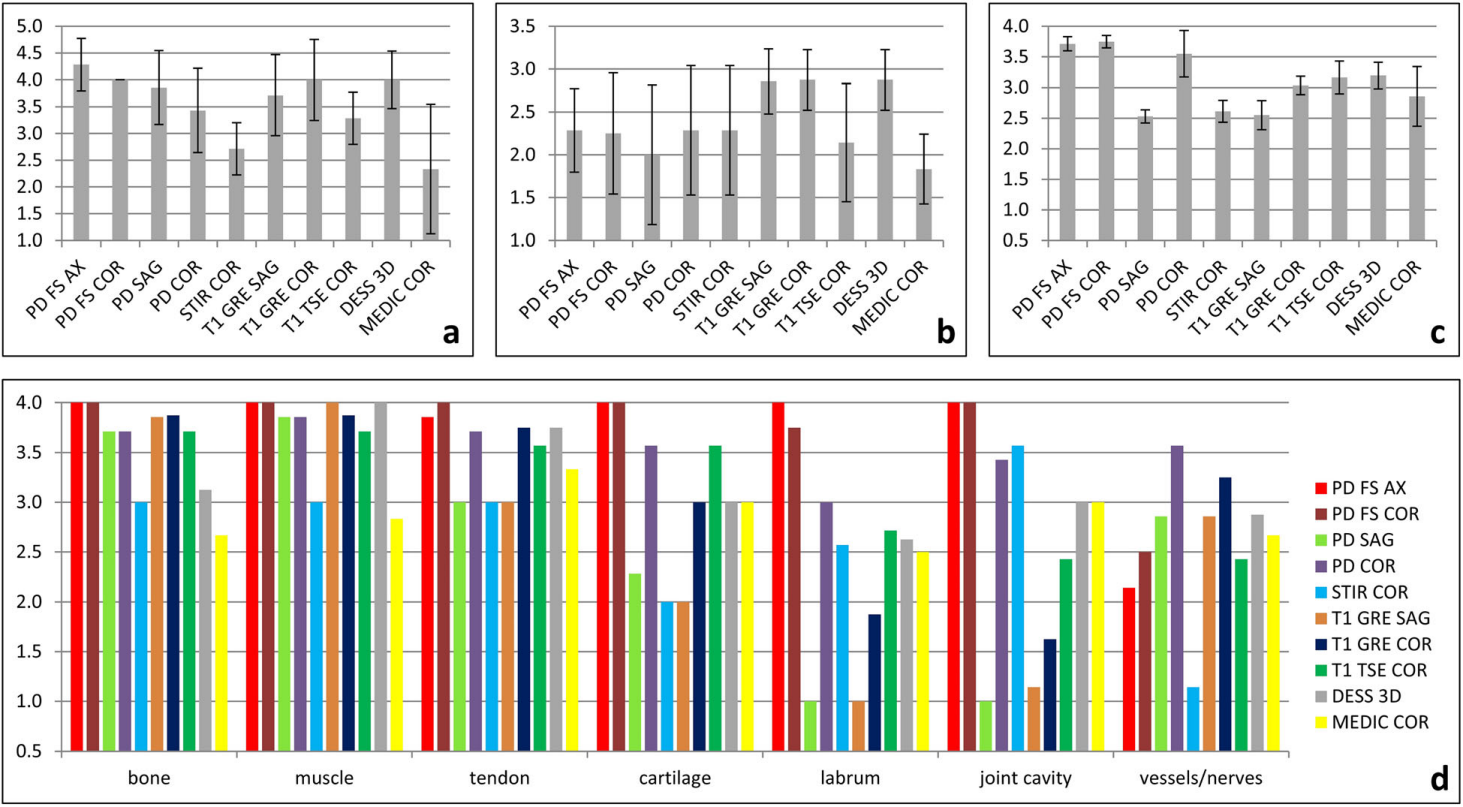
Image quality of 7-T shoulder MRI. The amount of artifacts (**a**), the intensity of B_1_^+^ inhomogeneities (**b**), and the structure assessability in detail for each structure (**d**) as well as averaged over all structures (**c**) is displayed for each sequence

Overall, the majority of the evaluated criteria for each sequence reached values above the median of each scale, especially when looking at the standard sequences. The amount of artifacts for each sequence is displayed in Fig. 1a and Table 2. Least artifacts were observed in PD fat-sat axial (scored 4.3 ± 0.5), followed by PD fat-sat coronal (scored 4.0 ± 0.0), T1 coronal (scored 4.0 ± 0.8), and DESS 3D (scored 4.0 ± 0.5). Severest artifacts appeared in MEDIC coronal (scored 2.3 ± 1.2 points) and STIR coronal (scored 2.7 ± 0.5 points). Results on the intensity of B_1_^+^ inhomogeneities for each sequence are reported in Fig. 1b and Table 2. The most homogenous B_1_^+^ field was observed for T1 sagittal, T1 COR, and 3D double-echo steady-state (DESS) (each of them scored 2.9 ± 0.4). The most inhomogeneities appeared in MEDIC coronal (scored 1.8 ± 0.4) and PD sagittal (scored 2.0 ± 0.8).

**Table 2:** Image quality evaluation

Sequence type	Artifacts (score 1–5)	B_1_^+^ inhomogeneities (score 1–3)	Assessability (score 1–4)
PD fat-sat TSE axial	4.3 ± 0.5	2.3 ± 0.5	3.7 ± 0.1
PD fat-sat TSE coronal	4.0 ± 0.0	2.3 ± 0.7	3.8 ± 0.1
PD TSE sagittal	3.9 ± 0.7	2.0 ± 0.8	2.5 ± 0.1
PD TSE coronal	3.4 ± 0.8	2.3 ± 0.8	3.6 ± 0.4
STIR coronal	2.7 ± 0.5	2.3 ± 0.8	2.6 ± 0.2
T1 GRE coronal	3.7 ± 0.8	2.9 ± 0.4	2.6 ± 0.2
T1 GRE coronal	4.0 ± 0.8	2.9 ± 0.4	3.0 ± 0.1
T1 TSE coronal	3.3 ± 0.5	2.1 ± 0.7	3.2 ± 0.3
DESS three-dimensional	4.0 ± 0.5	2.9 ± 0.4	3.2 ± 0.2
MEDIC coronal	2.3 ± 1.2	1.8 ± 0.4	2.9 ± 0.5

Results are displayed as mean ± standard deviation. *DESS* Dual-echo steady state, *Fat-sat* Fat-saturated, *GRE* Gradient echo, *MEDIC* Multi-echo data image combination, *PD* Proton density, *STIR* Short-tau inversion recovery, *TSE* Turbo-spin echo

Averaged over all structures (Fig. 1c, Table 2), the assessability was best in PD fat-sat coronal (scored 3.8 ± 0.1), PD fat-sat axial (scored 3.7 ± 0.1), and PD coronal (scored 3.6 ± 0.4) and worst in PD sagittal (scored 2.5 ± 0.1), T1 sagittal (scored 2.6 ± 0.2), and STIR coronal (scored 2.6 ± 0.2), in detail (Fig. 1d, Table 3), bone was best assessable in PD fat-sat coronal and axial (each of them scored 4.0 ± 0.0) and worst in MEDIC coronal (scored 2.7 ± 0.5) and STIR coronal (3.0 ± 0.0). Muscles were best assessable in PD fat-sat COR, PD fat-sat axial, DESS 3D, and T1 sagittal (each of them scored 4.0 ± 0.0 each) and worst in MEDIC coronal (scored 2.8 ± 0.8) and STIR coronal (scored 3.0 ± 0.0). Tendons were best assessable in PD fat-sat coronal (scored 4.0 ± 0.0) and PD fat-sat axial (scored 3.9 ± 0.4) and worst in T1 sagittal (scored 3.0 ± 0.6). Cartilage was best assessable in PD fat-sat coronal and axial (each of them scored 4.0 ± 0.0) and worst in STIR coronal (scored 2.0 ± 0.0) and T1 sagittal (scored 2.0 ± 0.8). The labrum was best assessable in PD fat-sat axial (scored 4.0 ± 0.0) and PD fat-sat coronal (scored 3.8 ± 0.5) and worst in PD sagittal and T1 sagittal (each of them scored 1.0 ± 0.0). The joint cavity was best assessable in PD fat-sat coronal and axial (each of them scored 4.0 ± 0.0) and worst in PD sagittal (scored 1.0 ± 0.0) and T1 sagittal (scored 1.1 ± 0.4). Vessels and nerves were best assessable in PD coronal (scored 3.6 ± 0.5) and T1 coronal (scored 3.3 ± 0.5) and worst in STIR coronal (scored 1.1 ± 0.4) and PD fat-sat axial (scored 2.1 ± 0.7).

**Table 3:** Structure assessability

Sequence type	Bone	Muscle	Tendon	Cartilage	Labrum	Joint cavity	Vessels/nerves
PD fat-sat TSE axial	4.0 ± 0.0	4.0 ± 0.0	3.9 ± 0.4	4.0 ± 0.0	4.0 ± 0.0	4.0 ± 0.0	2.1 ± 0.7
PD fat-sat TSE coronal	4.0 ± 0.0	4.0 ± 0.0	4.0 ± 0.0	4.0 ± 0.0	3.8 ± 0.5	4.0 ± 0.0	2.5 ± 0.5
PD TSE sagittal	3.7 ± 0.5	3.9 ± 0.4	3.0 ± 0.0	2.3 ± 0.5	1.0 ± 0.0	1.0 ± 0.0	2.9 ± 0.7
PD TSE coronal	3.7 ± 0.5	3.9 ± 0.4	3.7 ± 0.5	3.6 ± 0.5	3.0 ± 0.6	3.4 ± 0.8	3.6 ± 0.5
STIR coronal	3.0 ± 0.0	3.0 ± 0.0	3.0 ± 0.0	2.0 ± 0.0	2.6 ± 0.5	3.6 ± 0.5	1.1 ± 0.4
T1 GRE coronal	3.9 ± 0.4	4.0 ± 0.0	3.0 ± 0.6	2.0 ± 0.8	1.0 ± 0.0	1.1 ± 0.4	2.9 ± 0.7
T1 GRE coronal	3.9 ± 0.4	3.9 ± 0.4	3.8 ± 0.5	3.0 ± 0.5	1.9 ± 0.4	1.6 ± 0.5	3.3 ± 0.5
T1 TSE coronal	3.7 ± 0.5	3.7 ± 0.5	3.6 ± 0.5	3.6 ± 0.5	2.7 ± 0.5	2.4 ± 0.5	2.4 ± 0.5
DESS three-dimensional	3.1 ± 0.4	4.0 ± 0.0	3.8 ± 0.5	3.0 ± 0.0	2.6 ± 0.5	3.0 ± 0.8	2.9 ± 0.8
MEDIC coronal	2.7 ± 0.5	2.8 ± 0.8	3.3 ± 0.8	3.0 ± 0.6	2.5 ± 0.5	3.0 ± 0.6	2.7 ± 0.5

Results are displayed as mean ± standard deviation. The score from 1 to 4. *DESS* Dual-echo steady state, *Fat-sat* Fat saturated, *GRE* Gradient echo, *MEDIC* Multi-echo data image combination, *PD* Proton density, *STIR* Short-tau inversion recovery, *TSE* Turbo-spin echo

### Findings and diagnostic performance

With 7-T MRI, we identified pathologic findings regarding the rotator cuff in every patient. Predominantly, pathologies of the supraspinatus tendon (partial tears in seven patients, and tendinopathy in one patient), followed by tendinopathy of the subscapularis tendon (six patients) were observed. Two patients showed mild supraspinatus atrophy (plane geometry) with grade 1 fatty atrophy in one of them. Tendinopathy of the long bicipital tendon was observed in one patient. Degenerative changes of the acromioclavicular joint were seen in every patient (mild in six, severe in two patients). The glenohumeral cartilage appeared thinned in three patients and with a transchondral defect in one patient. Lesions of the labrum were diagnosed in three patients (two SLAP lesions and one Bankart lesion). No effusion was present in the glenohumeral joint of any patients. No bone marrow oedema was observed in the humeral head, the acromion, or the clavicle, but other bone marrow signal alterations (throughout cysts) were present in four patients. The acromion type was curved in two patients, flat in two patients, and convex in four patients. Pathologies of the subacromial subdeltoideal bursa were observed in five patients.

Every patient underwent shoulder arthroscopy after 7-T MRI. The delay between 7-T MRI and arthroscopy was from 2 and 15 days in seven patients (11.1 ± 4.5 days) and 70 days in one patient.

**Fig. 2:**
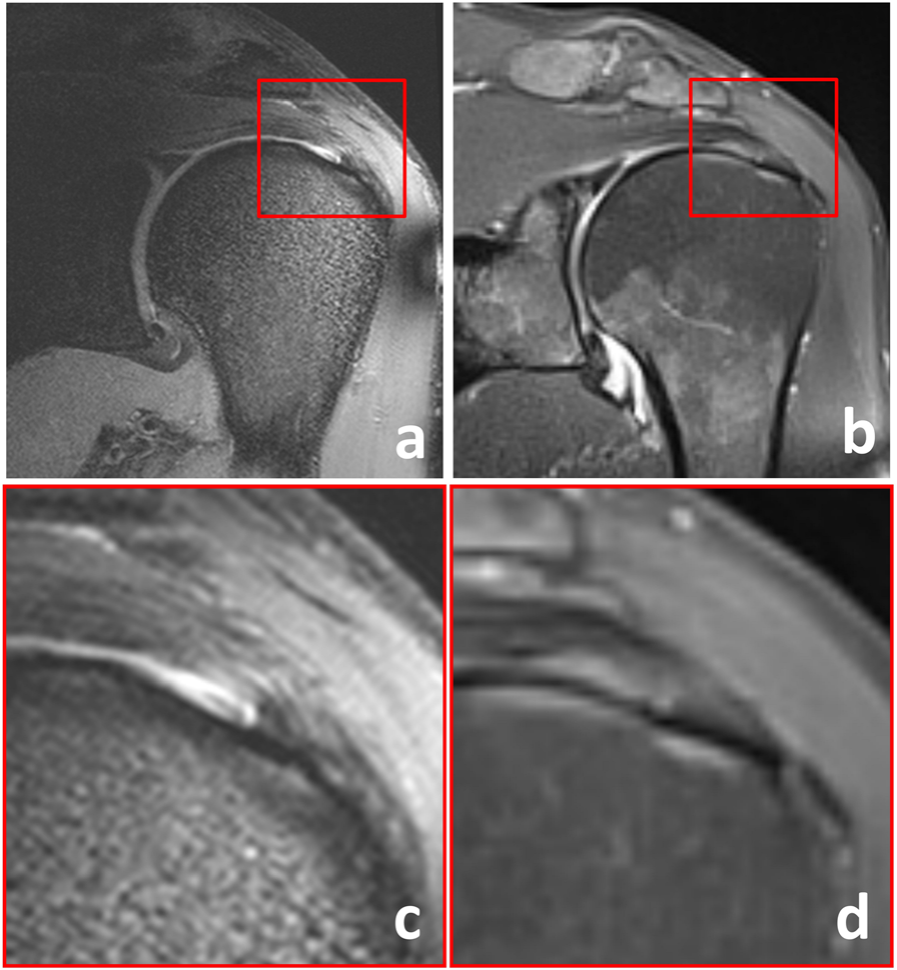
Example of overrating with 7-T MRI. In this 52-year-old male patient, a partial tear of the supraspinatus tendon was suspected according to 7-T MRI based on the signal increase and fluid collection near to the footprint (**a**, **c**). At 1.5 T (**b**, **d**), these changes are less impressive. Arthroscopy revealed little scar tissue and slight thinning of the tendon, but no tendinopathy or tear. Retrospectively, the signal changes observed at 7 T must be assigned to B_1_^+^ inhomogeneities and/or the magic angle effect

**Fig. 3:**
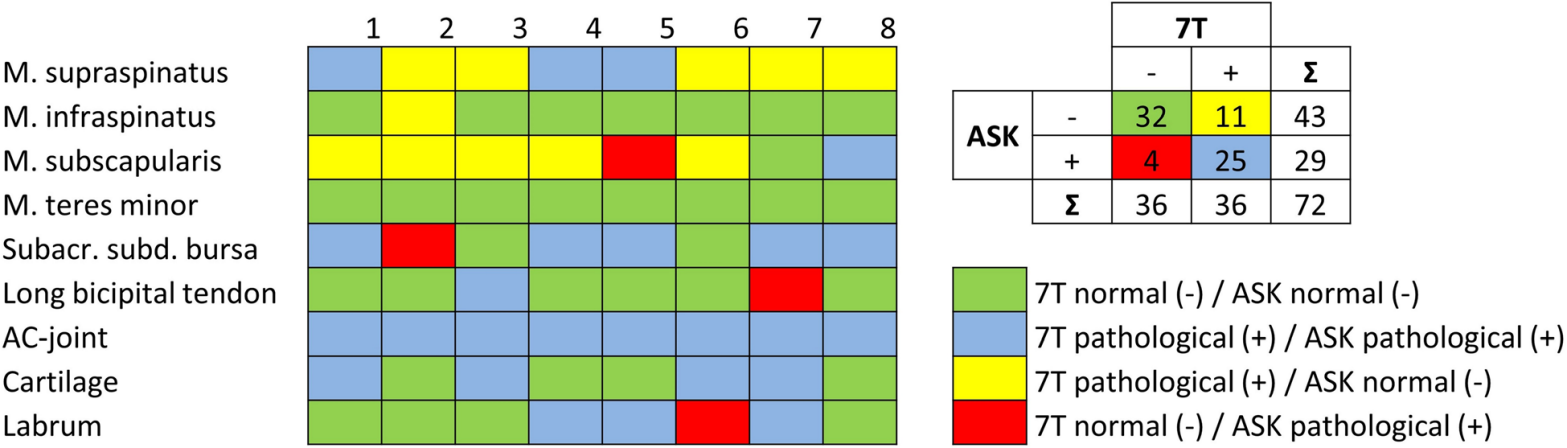
Correlation of pathologic findings reported at 7 T and arthroscopy (ASK) for patients 1–8. Green and blue fields indicate accordance between the modalities, whereas yellow fields represent overrating and red fields represent underrating with 7-T MRI

When compared to the arthroscopy report, pathologic findings of the rotator cuff tendons were widely overrated with 7 T MRI: arthroscopy did not confirm tendinopathy or partial tear of the supraspinatus tendon in five of eight patients, of the subscapularis tendon in five of six patients, of the infraspinatus tendon in one patient. An example of suspected supraspinatus tear on 7-T MRI, not confirmed by arthroscopy, is shown in Fig. 2. Only one pathologic finding of the rotator cuff (a partial lesion of the subscapularis tendon) according to arthroscopy was missed with 7-T MRI. Pathologic findings of the long bicipital tendon, the acromioclavicular joint, the glenohumeral cartilage, the labrum, and the subacromial subdeltoideal bursa were mainly concordant for 7-T MRI and arthroscopy. Exceptions were one lesion of the long bicipital tendon, one subacromial bursitis, and one SLAP lesion, missed on 7-T MRI. In Fig. 3, the results of 7-T MRI and arthroscopy are shown in comparison. Evaluating the results of all evaluated structures together, sensitivity, specificity, positive predictive value, and negative predictive value of 7-T MRI of the shoulder were 86% (95% confidence interval [CI] 68–96%), 74% (95% CI 59–86%), 69% (95% CI 57–79%), and 89% (95% CI 76–95%), respectively.

### Field strength comparison

**Fig. 4:**
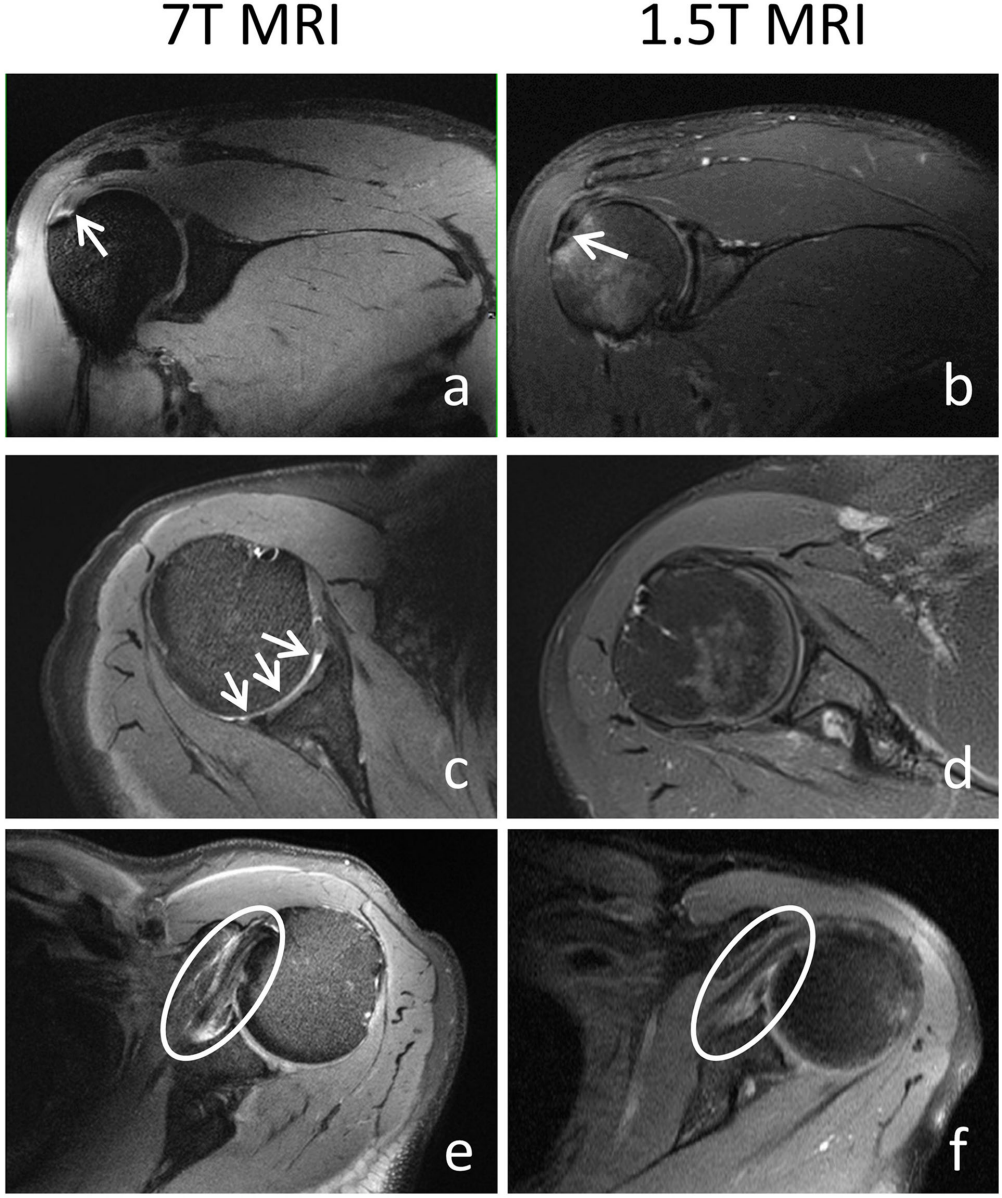
Pathologic findings associated with shoulder pain at 7 T and 1.5 T, confirmed by arthroscopy. Coronal proton density (PD)-weighted fat-saturated images of the right shoulder of a 31 year-old male patient, showing a partial rupture of the supraspinatus tendon (arrows): the partial tear near to the footprint of the tendon is more distinct at 7 T (**a**) than at 1.5 T (**b**) because of higher tissue contrast. Axial PD-weighted fat-saturated images of the right shoulder of a 51-year-old female patient with irregularities and thinning of the humeral cartilage: due to higher image resolution, pathology can clearly be depicted at 7 T (**c**, arrows), but not at 1.5 T (**d**). Axial PD-weighted fat-saturated images of the left shoulder of a 58-year-old male patient with tendinopathy of the subscapularis tendon (circle): the better tissue contrast and higher image resolution at 7 T (**e**) depicts thickening and oedema of the tendon much better compared to 1.5 T (**f**)

Pre-existing 1.5 T MRI images could be collected from four patients. The delay between the two examinations was 19, 34, 49, and 94 days, respectively. The main pathologies were visible at both field strengths. However, at 7 T, pathologies were more distinct due to better contrast and higher image resolution, as exemplarily shown in Fig. 4.

## Discussion

The evaluated 7-T shoulder MRI protocol outperforms the recommendations of the German Society of Radiology [21] regarding in-plane resolution and slice thickness and matches most of the recommendations regarding slice orientation and type of sequence. However, our protocol does not include a second echo (to obtain T2-weighted images) in the coronal PD fat-sat sequence, and the chosen echo times of the PD sequences of 30 and 35 ms, respectively, are slightly shorter than the recommendation of 40–60 ms, according to the recommendations of the European Society of Skeletal Radiology (Sports Sub-committee, 2016) [28]. A double echo and high TE are recommended to minimise the magic angle effect, which is responsible for artificial signal increase in structures orientated at 55° to the main magnetic field [29, 30]. In the shoulder, this artifact may lead to false positive diagnosis of supraspinatus tendinopathy [31]. Although we were aware of this pitfall, the sequence parameters were chosen intentionally to gain maximum SNR. As T2 relaxation time shortens with higher field strength, the magic angle effect gets less important at 7 T compared to 3 T and 1.5 T [32]. However, the magic angle effect might have led to the overrating of supraspinatus tendinopathy in our study even at 7 T. Therefore, we recommend to optimise the PD fat-sat sequences regarding higher TE and to implement a second echo for T2-weighting in further studies.

Regarding image quality and structure assessability, the parameters of the PD fat-sat sequences can be recommended at 7 T. T1-weighted gradient-echo sequences complement with their good B_1_^+^ homogeneity and support the assessment of bone and muscles. Although STIR yields good delineation of the joint cavity due to its high T2 contrast, this sequence provides images with more artifacts and stronger B_1_^+^ inhomogeneities, was inferior to PD fat-sat sequences, and should be deferred until substantial optimisation yields better results. Isotropic 3D imaging can be achieved by the use of a DESS sequence with excellent B_1_^+^ homogeneity, little artifacts, and good to moderate structure delineation. The MEDIC sequence was inferior to the other sequences regarding nearly all criteria and cannot be recommended.

After unblinding, we re-assessed the examinations where pathologies were missed at 7 T and looked for possible reasons. The partial tear of the subscapularis tendon was missed in the incomplete examination: among other sequences PD fat-sat axial could not be acquired due to a partial failure of the preamplifiers of the transmit/receive RF coil. The missed lesion of the long bicipital tendon and the missed subacromial bursitis could not be detected even retrospectively. In the first case, the delay between 7-T MRI and arthroscopy was 70 days. It is probable that the tendon lesion appeared during this delay. However, in the second case, the delay was only 14 days. The missed SLAP lesion could vaguely be discerned, retrospectively, but due to low signal-to-noise ratio and impaired image quality, it was not possible to make a 7-T MRI diagnosis in this particular patient.

Apart from the abovementioned magic angle effect, a psychological reason might have led to the overrating of tendinopathies in our study: the likelihood of tendon pathologies due to the patient selection criteria combined with the expectation of a high level of detail at 7 T might have influenced the readers to over-interpret signal changes and biased the radiological report [33]. Therefore, a blinded setting including healthy volunteers and different field strengths is desirable for future studies.

So far, the diagnostic accuracy of 7-T shoulder MRI is inferior to the results of 3 T studies published in the literature. In the detection of supraspinatus tears at 3 T, Mc Garvey et al. [34] reported in their meta-analysis sensitivities of 85.7% and 80.5% and specificities of 99% and 100% for full- and partial-thickness tears, respectively. However, a high number of false positive results was reported in patients with adhesive capsulitis, where MRI predicted a rotator cuff pathology in 57.9 % while arthroscopy revealed an incidence of only 13.2% [35]. In the context of MR arthrography, 7-T shoulder MRI can aid with its high resolution and high contrast of the joint fluid. However, without expansion of the joint cavity, even 7-T might stay inferior to lower field strength MR arthrography, especially in the detection of cartilage and labrum lesions. To further assess the diagnostic accuracy of 7-T shoulder MRI in detail and to make a statistical analysis more reliable, a subsequent study with a higher number of patients is needed. In addition, 3-T scans of every patient are desirable for direct comparison as well as a comparative analysis to 3-T MR arthrography.

In summary, MRI of the shoulder with diagnostic image quality is feasible at 7 T. Although the diagnostic accuracy still did not reach the reported results of 3-T MRI, yet, our study marks the way to implement shoulder imaging in the scope of clinical 7-T MRI applications. Even at ultra-high field, readers should be aware of the magic angle effect and in particular of the bias to over-interpret signal alterations.

## Data Availability

The datasets used and/or analysed during the current study are available from the corresponding author on reasonable request.

## Abbreviations

3D: Three-dimensional
DESS: Dual echo steady-state
Fat-sat: Fat-saturated
GRE: Gradient echo
MEDIC: Multi-echo data image combination
MRI: Magnetic resonance imaging
PD: Proton density
RF: Radiofrequency
SD: Standard deviation
SLAP: Superior glenoid labrum anterior to posterior
STIR: Short-tau inversion recovery
TSE: Turbo-spin echo

## References

[1] Major N, Morrison WB, Coker D (2015). The shoulder. Top Magn Reson Imaging..

[2] Gottsegen CJ, Merkle AN, Bencardino JT, Gyftopoulos S (2017). Advanced MRI techniques of the shoulder joint: current applications in clinical practice. AJR Am J Roentgenol..

[3] Sharma G, Bhandary S, Khandige G, Kabra U (2017). MR imaging of rotator cuff tears: correlation with arthroscopy. J Clin Diagn Res..

[4] Polster JM, Schickendantz MS (2010). Shoulder MRI: what do we miss?. AJR Am J Roentgenol..

[5] VanBeek C, Loeffler BJ, Narzikul A (2014). Diagnostic accuracy of noncontrast MRI for detection of glenohumeral cartilage lesions: a prospective comparison to arthroscopy. J Shoulder Elbow Surg..

[6] Yildiz F, Bilsel K, Pulatkan A, Uzer G, Aralasmak A, Atay M (2017). Reliability of magnetic resonance imaging versus arthroscopy for the diagnosis and classification of superior glenoid labrum anterior to posterior lesions. Arch Orthop Trauma Surg..

[7] Karamat MI, Darvish-Molla S, Santos-Diaz A (2016). Opportunities and challenges of 7 Tesla magnetic resonance imaging: a review. Crit Rev Biomed Eng..

[8] Daniell H, Geere JA, Toms AP, Hing CB, Smith TO (2012). The diagnostic accuracy of MRI for the detection of partial- and full-thickness rotator cuff tears in adults. Magn Reson Imaging..

[9] Obusez EC, Lowe M, Oh SH (2018). 7 T MR of intracranial pathology: preliminary observations and comparisons to 3 T and 1.5 T. Neuroimage..

[10] Goebel J, Nensa F, Schemuth HP (2018). Feasibility of aortic valve planimetry at 7 T ultrahigh field MRI: comparison to aortic valve MRI at 3 T and 1.5 T. Eur J Radiol Open..

[11] Lazik-Palm A, Kraff O, Johst S (2016). Morphological and quantitative 7 T MRI of hip cartilage transplants in comparison to 3 T-initial experiences. Invest Radiol..

[12] Noebauer-Huhmann IM, Szomolanyi P, Kronnerwetter C (2015). Brain tumours at 7 T MRI compared to 3 T--contrast effect after half and full standard contrast agent dose: initial results. Eur Radiol..

[13] Beiderwellen K, Kraff O, Laader A (2017). Contrast enhanced renal MR angiography at 7 Tesla: how much gadolinium do we need?. Eur J Radiol..

[14] Kraff O, Quick HH (2017). 7 T: Physics, safety, and potential clinical applications. J Magn Reson Imaging..

[15] U.S. Food & Drug Administration (2017) FDA clears first 7 T magnetic resonance imaging device. Available via https://www.fda.gov/news-events/press-announcements/fda-clears-first-7t-magnetic-resonance-imaging-device. Accessed 22 Oct 2019.

[16] Krug R, Stehling C, Kelley DA, Majumdar S, Link TM (2009). Imaging of the musculoskeletal system in vivo using ultra-high field magnetic resonance at 7 T. Invest Radiol..

[17] Kraff O, Quick HH (2019). Radiofrequency coils for 7 Tesla MRI. Top Magn Reson Imaging..

[18] Brown R, Deniz CM, Zhang B, Chang G, Sodickson DK, Wiggins GC (2014). Design and application of combined 8-channel transmit and 10-channel receive arrays and radiofrequency shimming for 7-T shoulder magnetic resonance imaging. Invest Radiol..

[19] Rietsch SHG, Pfaffenrot V, Bitz AK (2017). An 8-channel transceiver 7-channel receive RF coil setup for high SNR ultrahigh-field MRI of the shoulder at 7 T. Med Phys..

[20] Kraff O, Bitz AK, Dammann P, Ladd SC, Ladd ME, Quick HH (2010). An eight-channel transmit/receive multipurpose coil for musculoskeletal MR imaging at 7 T. Med Phys..

[21] (2018) Protokollempfehlungen der AG Bildgebende Verfahren des Bewegungsapparates (AG BVB) der Deutschen Röntgengesellschaft (DRG) zu Messsequenzen für die Gelenk-MRT. Rofo. 190:186–190. 10.1055/s-0043-125412 10.1055/s-0043-125412 29346816

[22] Theysohn JM, Kraff O, Orzada S (2013). Bilateral hip imaging at 7 Tesla using a multi-channel transmit technology: initial results presenting anatomical detail in healthy volunteers and pathological changes in patients with avascular necrosis of the femoral head. Skeletal Radiol..

[23] Sutter R, Hodek R, Fucentese SF, Nittka M, Pfirrmann CW (2013). Total knee arthroplasty MRI featuring slice-encoding for metal artifact correction: reduction of artifacts for STIR and proton density-weighted sequences. AJR Am J Roentgenol..

[24] Park JE, Choi YH, Cheon JE (2018). Three-dimensional radial VIBE sequence for contrast-enhanced brain imaging: an alternative for reducing motion artifacts in restless children. AJR Am J Roentgenol..

[25] Lazik A, Landgraeber S, Schulte P, Kraff O, Lauenstein TC, Theysohn JM (2015). Usefulness of metal artifact reduction with WARP technique at 1.5 and 3 T MRI in imaging metal-on-metal hip resurfacings. Skeletal Radiol..

[26] Tawfik AM, El-Morsy A, Badran MA (2014). Rotator cuff disorders: how to write a surgically relevant magnetic resonance imaging report?. World J Radiol..

[27] Somerson JS, Hsu JE, Gorbaty JD, Gee AO (2016). Classifications in brief: Goutallier classification of fatty infiltration of the rotator cuff musculature. Clin Orthop Relat Res..

[28] European Society of Skeletal Radiology (Sports Sub-committee) (2016) Guidelines for MR imaging of sports injuries. Available via https://www.essr.org/content-essr/uploads/2016/10/ESSR-MRI-Protocols-Shoulder.pdf. Accessed 22 Oct 2019.

[29] Erickson SJ, Cox IH, Hyde JS, Carrera GF, Strandt JA, Estkowski LD (1991). Effect of tendon orientation on MR imaging signal intensity: a manifestation of the “magic angle” phenomenon. Radiology..

[30] Hayes CW, Parellada JA (1996). The magic angle effect in musculoskeletal MR imaging. Top Magn Reson Imaging..

[31] Madden ME (2006). The magic-angle effect of the supraspinatus tendon. Radiol Technol..

[32] Gold GE, Suh B, Sawyer-Glover A, Beaulieu C (2004). Musculoskeletal MRI at 3.0 T: initial clinical experience. AJR Am J Roentgenol..

[33] Michael Robert B., Garry Maryanne, Kirsch Irving (2012). Suggestion, Cognition, and Behavior. Current Directions in Psychological Science.

[34] McGarvey Ciaran, Harb Ziad, Smith Christian, Houghton Russell, Corbett Steven, Ajuied Adil (2015). Diagnosis of rotator cuff tears using 3-Tesla MRI versus 3-Tesla MRA: a systematic review and meta-analysis. Skeletal Radiology.

[35] Loeffler BJ, Brown SL, D'Alessandro DF, Fleischli JE, Connor PM (2011). Incidence of false positive rotator cuff pathology in MRIs of patients with adhesive capsulitis. Orthopedics..

